# DNA barcoding of morphologically characterized mosquitoes belonging to the subfamily Culicinae from Sri Lanka

**DOI:** 10.1186/s13071-018-2810-z

**Published:** 2018-04-25

**Authors:** Thilini Chathurika Weeraratne, Sinnathamby Noble Surendran, S. H. P. Parakrama Karunaratne

**Affiliations:** 1 Department of Zoology, Faculty of Science, University of Peradeniya, Peradeniya, Sri Lanka; 2 Department of Zoology, Faculty of Science, University of Jaffna, Jaffna, Sri Lanka; 3 National Institute of Fundamental Studies, Hantana, Kandy, Sri Lanka

**Keywords:** DNA barcoding, Culicinae, Mosquitoes, *Aedes*, *Culex*, Sri Lanka, *cox*1, ITS2

## Abstract

**Background:**

Vectors of mosquito-borne diseases in Sri Lanka, except for malaria, belong to the subfamily Culicinae, which includes nearly 84% of the mosquito fauna of the country. Hence, accurate and precise species identification of culicine mosquitoes is a crucial factor in implementing effective vector control strategies. During the present study, a combined effort using morphology and DNA barcoding was made to characterize mosquitoes of the subfamily Culicinae for the first time from nine districts of Sri Lanka. Cytochrome *c* oxidase subunit 1 (*cox*1) gene from the mitochondrial genome and the internal transcribed spacer 2 (ITS2) region from the nuclear ribosomal DNA were used for molecular characterization.

**Results:**

According to morphological identification, the field collected adult mosquitoes belonged to 5 genera and 14 species, i.e. *Aedes aegypti*, *Ae. albopictus*, *Ae. pallidostriatus*, *Aedes* sp. 1, *Armigeres* sp. 1, *Culex bitaeniorhynchus*, *Cx. fuscocephala*, *Cx. gelidus*, *Cx. pseudovishnui*, *Cx. quinquefasciatus*, *Cx. tritaeniorhynchus*, *Cx. whitmorei*, *Mansonia uniformis* and *Mimomyia chamberlaini*. Molecular analyses of 62 *cox*1 and 36 ITS2 sequences were exclusively comparable with the morphological identifications of all the species except for *Ae. pallidostriatus* and *Aedes* sp. 1. Although the species identification of *Armigeres* sp. 1 specimens using morphological features was not possible during this study, DNA barcodes of the specimens matched 100% with the publicly available *Ar. subalbatus* sequences, giving their species status. Analysis of all the *cox*1 sequences (14 clades supported by strong bootstrap value in the Neighbor-Joining tree and interspecific distances of > 3%) showed the presence of 14 different species. This is the first available DNA sequence in the GenBank records for morphologically identified *Ae. pallidostriatus*. *Aedes* sp. 1 could not be identified morphologically or by publicly available sequences. *Aedes aegypti*, *Ae. albopictus* and all *Culex* species reported during the current study are vectors of human diseases. All these vector species showed comparatively high diversity.

**Conclusions:**

The current study reflects the significance of integrated systematic approach and use of *cox*1 and ITS genetic markers in mosquito taxonomy. Results of DNA barcoding were comparable with morphological identifications and, more importantly, DNA barcoding could accurately identify the species in the instances where the traditional morphological identification failed due to indistinguishable characters of damaged specimens and the presence of subspecies.

**Electronic supplementary material:**

The online version of this article (10.1186/s13071-018-2810-z) contains supplementary material, which is available to authorized users.

## Background

Correct species recognition of mosquito vectors is a vital component in implementing effective vector control strategies. Morphology based taxonomy and molecular characterization are the two major approaches used in species identification. Taxonomic keys used in morphological identification of mosquitoes mainly depend on the external features of adult females and fourth-instar larvae where the specimens must be handled and stored cautiously without damaging the external features, which is not practically possible all the time. Moreover, this approach needs expertise and is time consuming. Also, it does not identify the factors such as genetic variations and phenotypic plasticity that can have an impact on species level identification [1]. Alternatively, DNA barcoding or molecular characterization has become increasingly popular as an efficient method of species identification since it produces results with high precision even from a part of the specimen, within a short period of time [1]. Molecular taxonomy enables researchers to identify mosquitoes to species or subspecies level, understand genetic diversity and make predictions on evolution and phylogenetic relationships. Among all the barcoded insect groups, mosquitoes are the most intensely barcoded [2], probably because of their importance as vectors of many life threatening human diseases.

A region of the cytochrome *c* oxidase subunit 1 (*cox*1) gene located in the mitochondrial genome and a region in the nuclear ribosomal DNA internal transcribed spacer 2 (ITS2) have been the most frequently used genetic markers in DNA barcoding of mosquitoes. *cox*1 has been used as the only molecular marker in characterizing 37 Canadian, 62 Indian, 122 Chinese, 32 Pakistani and 45 Singaporean mosquito species [3–7]. A study conducted in India has used the same marker in investigating the molecular evolution of mosquito vectors of medical and veterinary importance [8]. The ITS2 region has been used in distinguishing closely related mosquito species belonging to various genera of the subfamily Culicianae, such as *Culex* [9] and *Aedes* [10]. Another barcode region used in mosquito barcoding studies is *cox*2 of the mitochondrial genome [11, 12]. D3 expansion segment (~1000 bp) a coding region located in the large subunit of the nuclear ribosomal DNA is also used as a molecular marker in mosquito barcoding studies since it shows a higher inter-/intraspecific variations.

Molecular markers, as a group, will provide more reliable information on the genetic variation between and within a species. Sri Lankan anophelines that included major and potential vectors of malaria have been studied successfully using both *cox*1 and ITS2 markers [13]. A study has used both these marker regions in barcoding the vector mosquitoes *Ae. aegypti*, *Ae. albopictus*, *Culex tritaeniorhynchus*, *Cx. vishnui* and *Cx. pseudovishnui* [14]. A combination of *cox*1, ITS2 and D3 has been used in another study to recognize sibling species of several mosquito species complexes [15]. However, researchers state that characterization of mosquitoes through integrated approaches using both morphological and molecular identification is the best approach for species identification [5].

Sri Lanka is a tropical country with high mosquito diversity supported by both natural and man-made factors for breeding and survival of mosquitoes. A total of 141 mosquito species belonging to 17 genera and two subfamilies have been reported from the country [16–18]. Among these 17 genera, *Anopheles* (23 species) belongs to the subfamily Anophelinae and the rest, i.e. *Aedeomyia* (1 species), *Aedes* (48 species), *Armigeres* (4 species), *Coquillettidia* (1 species), *Culex* (37 species), *Ficalbia* (1 species), *Heizmannia* (1 species), *Hodgesia* (2 species), *Malaya* (1 species), *Mansonia* (3 species), *Mimomyia* (4 species), *Orthopodomyia* (2 species), *Topomyia* (2 species), *Toxorhynchites* (2 species), *Tripteroides* (3 species) and *Uranotaenia* (7 species), belong to the subfamily Culicinae [16–18]. Culicinae is the largest group in Sri Lanka, represented by 118 species (~84% of the total mosquito species in the country). Mosquito-borne diseases have adversely affected the economy and the public health of the country. Nearly 150,000 dengue cases and 300 deaths by dengue have been recorded for the first eight months of 2017 [19]. Dengue and chikungunya are transmitted by *Aedes aegypti* and *Ae. albopictus* [20, 21]*. Culex tritaeniorhynchus* and *Cx. gelidus* transmit Japanese encephalitis [22], whereas *Cx. quinquefasciatus* transmits filariasis [23]. *Anopheles culicifacies* and *An. subpictus* act as the primary and secondary vectors, respectively, of malaria in Sri Lanka [24, 25]. Previously we reported molecular characterization of 16 anopheline species from Sri Lanka [13]. Here were report characterization of culicinae mosquitoes collected from nine administrative districts of Sri Lanka, using both morphology and molecular based taxonomy. Two commonly used genetic markers, cox1and ITS2, were used in molecular characterization.

## Methods

### Study sites

**Fig. 1:**
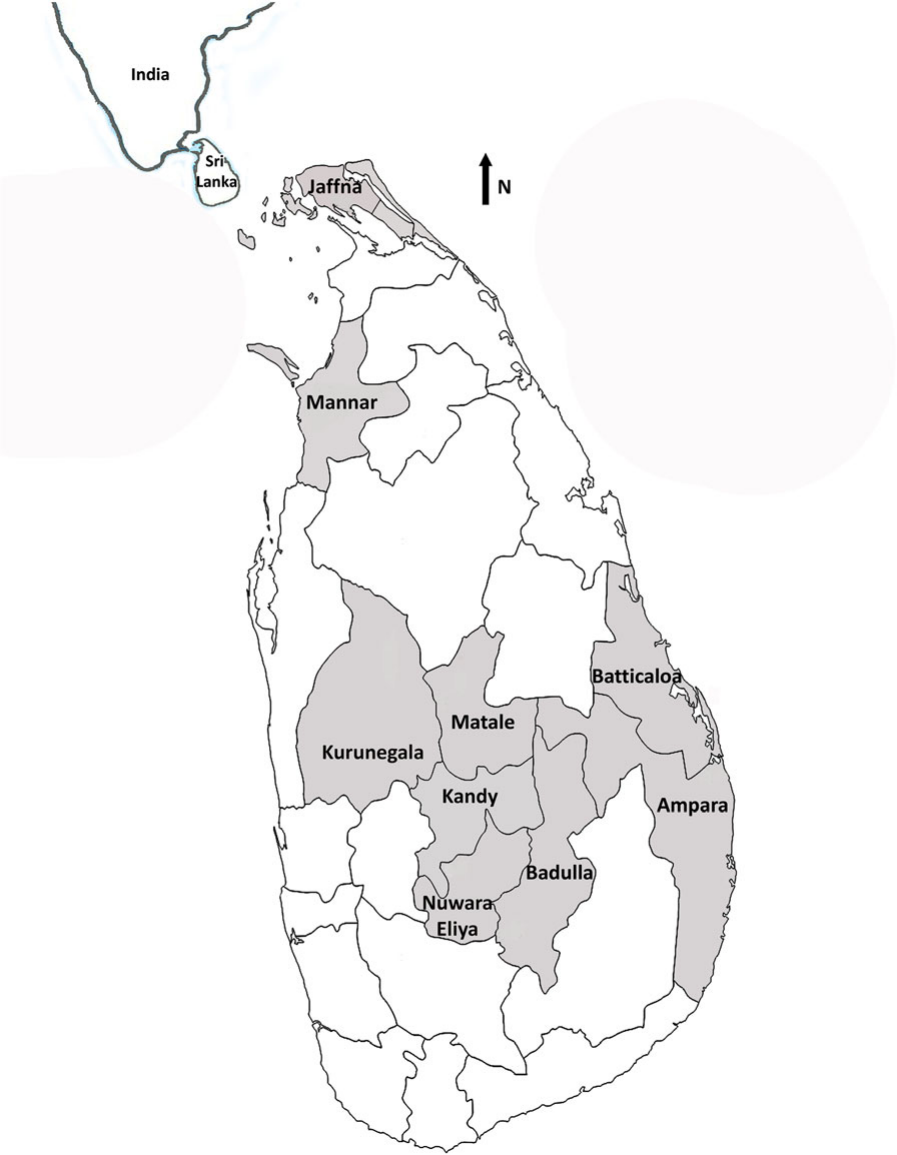
Map of Sri Lanka showing the nine administrative districts from which the mosquitoes were collected for the study

Mosquitoes were collected from nine administrative districts located in the three main climatic zones in Sri Lanka, i.e. Kandy, Matale and Nuwara-Eliya in wet zone (annual rainfall > 2500 mm rainfall); Badulla and Kurunegala in the intermediate zone (annual rainfall 1750–2500 mm rainfall) and Ampara, Batticaloa, Jaffna and Mannar in dry zone (annual rainfall < 1750 mm rainfall) Ampara, Batticaloa and Jaffna were coastal areas covered with little vegetation, few paddy fields and few human settlements. Kurunegala had little vegetation but a river running along the collection site. A dense vegetation cover was observed in the vicinity of Badulla and Matale study sites. Kandy site had a moderate vegetation cover and was surrounded by a significant human settlement. Mannar site was largely surrounded by paddy fields with few human settlements (Fig. 1).

### Mosquito collection

Adult mosquitoes were collected using cattle baited trap-huts, backpack aspirators, light traps and BG Sentinel traps. Dried specimens (a minimum of 10 individual mosquitoes) were used in morphological and molecular identifications. Mosquitoes were morphologically identified to generic and species level using standard taxonomic keys [26–29]. Voucher specimens of each species were pin mounted for a reference collection at the Invertebrate Systematic Diversity Facility (ISDF) at the Department of Zoology, Faculty of Science, University of Peradeniya, Peradeniya, Sri Lanka (UP/ZOO/BT011 - KU015). The reminder were dried and stored for molecular characterization.

### DNA extraction, polymerase chain reaction (PCR) and sequencing

Genomic DNA was extracted from head and thoracic regions of each individual using following the method described by Livak [30]. Cytochrome *c* oxidase subunit 1 (*cox*1) gene was amplified using forward primer C1-J-1718 (5'-GGA GGA TTT GGA AAT TGA TTA GTT CC-3') and reverse primer C1-N-2191 (5'-CCC GGT AAA ATT AAA ATA TAA ACT TC-3') [31]. ITS2 region was amplified using the forward primer 5'-ATC ACT CGG CTC ATG GAT CG-3' and the reverse primer 5'-ATG CTT AAA TTT AGG GGG TAG TC-3' [32]. Each amplification was performed in 15 μl that included 1 μl of DNA template, 1.5 μl of 10× KAPA buffer A (Cape Town, South Africa), 0.12 μl of KAPA Taq, 0.12 μl of 2.5 mM dNTP mix, 0.75 μl of 50 mM MgCl_2_, 0.51 μl of each primer (10 mmol) and 10.49 μl of ddH_2_O. PCR parameters were 95 °C for 5 min and 35 cycles of 94 °C for 30 s, 51 °C (for *cox*1) / 55 °C (for ITS2) for 40 s and 72 °C for 45 s, followed by a final extension step of 72 °C for 10 min. PCR products were run in 1.5% agarose gel stained with ethidium bromide and visualized in a gel imaging system.

PCR products showing positive clear bands were purified using QIAquick® PCR Purification kits (Hilden, Germany) according to the manufacturers’ protocol. A maximum of three PCR positive samples of each species were sequenced bi-directionally at the Department of Molecular Biology and Biotechnology, Faculty of Science, University of Peradeniya.

### DNA sequence analysis

The trace files/chromatograms of *cox*1 and ITS2 sequences were manually edited using BioEdit software. Sequences of low quality were excluded during data analysis. Consensus sequences were aligned using Clustal W in BioEdit software. Once the alignment was completed, species identification was confirmed by comparison to publicly available sequence data in GenBank using Basic Logical Alignment Search Tool (BLAST) [https://blast.ncbi.nlm.nih.gov/Blast.cgi] and the Barcode of Life Database (BOLD) interface [www.boldsystems. org]. The number of parsimony informative sites, number of variable sites, number of haplotypes, haplotype diversity and GC content were analyzed using the DNA Sequences Polymorphism software (DnaSP, version 5.1.10). MEGA version 6.0 was used to calculate intraspecific and interspecific pairwise sequence divergence using the Kimura-2 parameter distance model [33]. Neighbor-Joining (NJ) phylogenetic trees of *cox*1 and ITS2 sequences were constructed in MEGA 6.0 using Kumura-2 Parameter distances. Branch support of NJ trees were assessed by bootstrapping with 1000 replicates. Codon positions included 1st + 2nd + 3rd + noncoding regions. All the haplotype sequences of *cox*1 and ITS2 were deposited in the GenBank database (see below).

## Results

According to morphological identification, the mosquitoes collected belonged to 14 species of 5 genera *Aedes*, *Armigeres*, *Culex*, *Mansonia* and *Mimomyia* (Table 1). A total of 62 *cox*1 sequences obtained from all 14 species and 36 ITS2 sequences obtained from only 10 species were used in genetic diversity and phylogenetic analysis. The fragment sizes of *cox*1 was 428 bp and that of ITS2 sequences ranged from 335 bp (*Ae. aegypti*) to 403 bp (*Ae. albopictus*) (Table 1). ITS2 sequences generated for *Cx. bitaeniorhynchus*, *Cx. fuscocephala*, *Cx. gelidus* and *Cx. whitmorei* were not considered for analysis as they were not of good quality. Fifty *cox*1 and 29 ITS2 haplotypes were obtained (Table 1).

**Table 1:** The intraspecific distances and haplotype diversity of *cox*1 sequences. Intraspecific distances were calculated using Kimura 2-Parameter distance algorithm

Species	*n*	h	GenBank accession numbers	Mean distance	Pairwise distance range	Haplotype diversity
*Aedes aegypti* (Linnaeus, 1762)	4	3	KY352243–KY352244, KY352256	0.010 ± 0.004	0.000–0.019	0.833 ± 0.049
*Aedes albopictus* (Skuse, 1894)	6	3	KY352245–KY352247	0.002 ± 0.001	0.000–0.005	0.600 ± 0.046
*Aedes pallidostriatus* (Theobald, 1907)	6	4	KY352248–KY352251	0.009 ± 0.003	0.000–0.017	0.800 ± 0.030
*Aedes* sp. 1	4	4	KY352252–KY352255	0.003 ± 0.002	0.002–0.005	1.000 ± 0.031
*Armigeres subalbatus* (Coquillett, 1898)	9	7	KY352257–KY352263	0.004 ± 0.002	0.000–0.007	0.944 ± 0.005
*Culex bitaeniorhynchus* (Giles, 1901)	1	1	KY040659	n/c	–	n/c
*Culex fuscocephala* (Theobald, 1907)	2	2	KY040660–KY040661	0.014 ± 0.006	–	1.000 ± 0.250
*Culex gelidus* (Theobald, 1901)	2	1	KY053491	n/c	–	n/c
*Culex pseudovishnui* (Colless, 1957)	4	4	KY040662–KY040665	0.009 ± 0.004	0.005–0.012	1.000 ± 0.016
*Culex quinquefasciatus* (Say, 1823)	4	4	KY040666–KY040669	0.002 ± 0.002	0.000–0.005	1.000 ± 0.050
*Culex tritaeniorhynchus* (Giles, 1901)	5	5	KY040670–KY040674	0.007 ± 0.002	0.002–0.012	1.000 ± 0.031
*Culex whitmorei* (Giles, 1904)	1	1	KY040675	n/c	–	n/c
*Mansonia uniformis* (Theobald, 1901)	11	9	KY352264–KY352272	0.008 ± 0.003	0.000–0.017	0.945 ± 0.004
*Mimomyia chamberlaini* (Ludlow, 1904)	3	2	KY352273–KY352274	0.002 ± 0.002	0.000–0.002	0.667 ± 0.099

*Abbreviations*: *n* total number of *cox*1 sequences, *h* number of haplotypes

Species identifications were performed using *cox*1 and ITS2 sequences compared to already available sequences in GenBank and BOLD system (Additional file 1: Table S1). Sequences obtained for *Aedes aegypti*, *Aedes albopictus*, all 7 *Culex* species, *Mansonia uniformis* and *Mimomyia chamberlaini* were 100% corresponding with their morphological species identifications. *cox*1 and ITS2 sequences generated for morphologically identified *Armigeres* sp. 1 specimens showed 99% similarity to the publicly available *Armigeres subalbatus* sequences, confirming its distinct species status.

Sequence data for *cox*1 and ITS2 markers from *Aedes pallidostriatus* were not available in the GenBank or BOLD systems to compare the sequence data generated for the morphologically identified *Aedes pallidostriatus*. The closest available sequences were from *Aedes ochraceus* (92% *cox*1 sequence similarity and 90% ITS2 similarity). *Aedes* sp. 1 could not be identified using taxonomic keys due to damages in identification features in the samples collected. According to molecular data, *Ae. vexans* was the closest species to *Aedes* sp. 1 specimens, with a *cox*1 similarity of 94% and ITS2 similarity of 82%. Hence, the species level identification of *Aedes* sp. 1 could not be confirmed using the available morphological and molecular data.

### Phylogenetic analysis

**Fig. 2:**
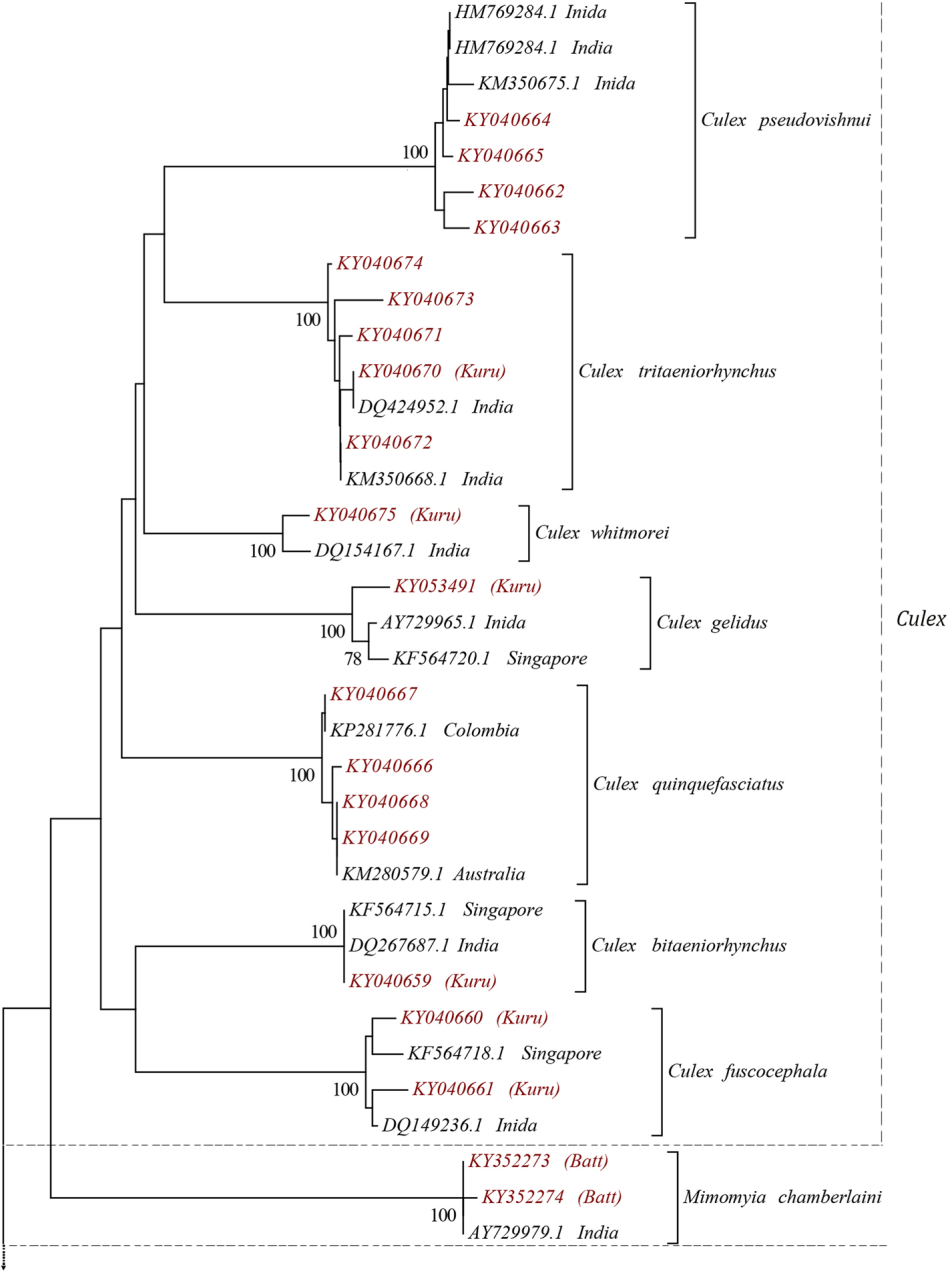
NJ phylogenetic tree (based on Kimura 2-parameter genetic distance model) based on *cox*1 sequences of all the 50 haplotypes of 14 species belonging to the subfamily Culicinae, collected from Sri Lanka during the study (red labels) and the sequences retrieved from the GenBank database (back labels). Only nodal support > 70% is shown

**Fig. 3:**
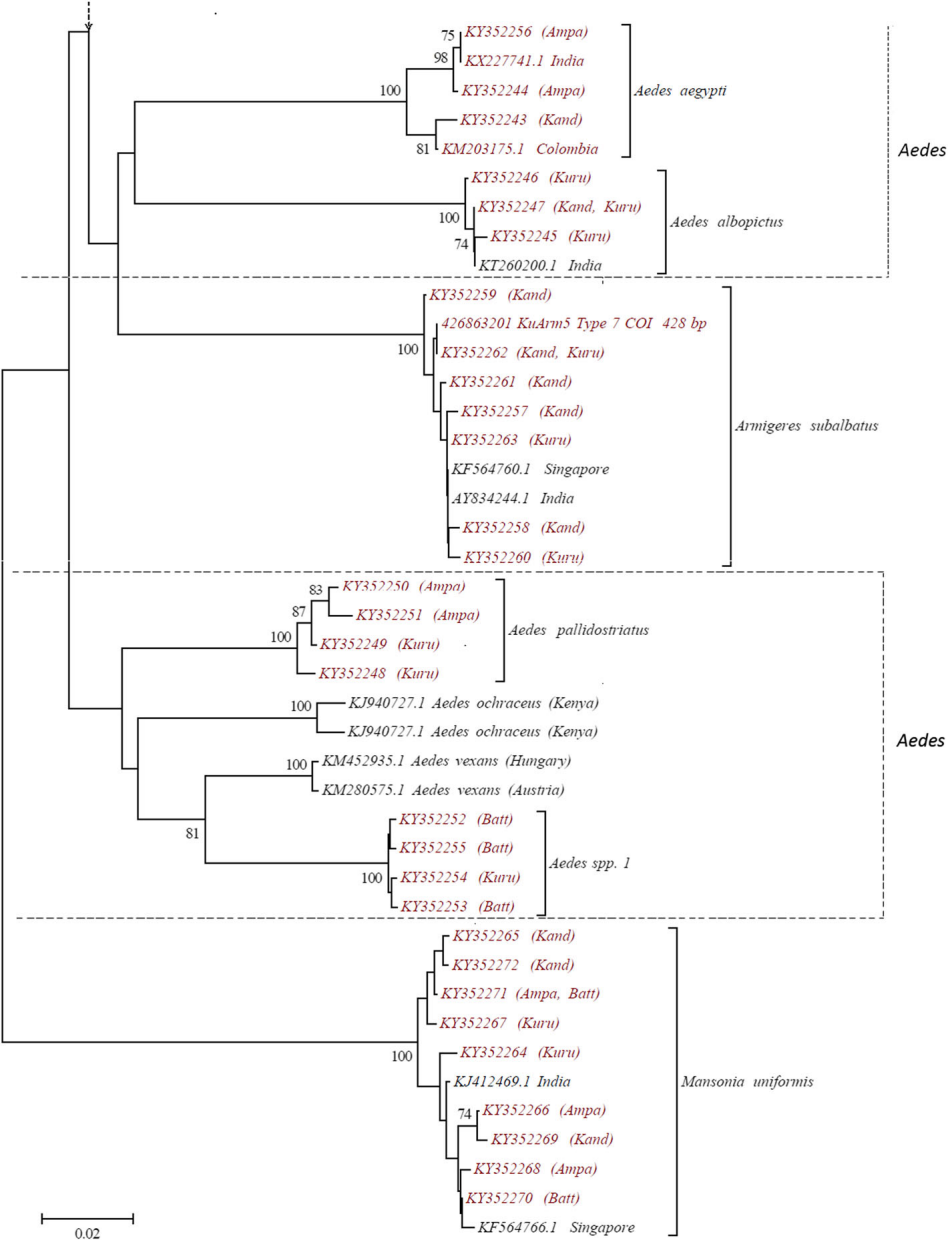
NJ phylogenetic tree based on Kimura 2-parameter genetic distance model for *cox*1 sequences of all the 50 haplotypes of 14 species belonging to the subfamily Culicinae, collected from Sri Lanka during the study (red labels) and the sequences retrieved from the GenBank database (back labels); continuation of Fig.2. Only nodal support > 70% is shown

The NJ tree constructed using the *cox*1 sequences formed 14 strongly supported clades (bootstrap value of 100%) 100% compatible with morphological identification (Figs. 2 and 3). All 7 species of genus *Culex* clustered together with *Mi. chamberlaini* specimens to form a single major clade, highlighting their close relationship. These two genera also share many common morphological characters. Haplotypes of *Aedes aegypti* and *Ae. albopictus* formed a separate clade while *Ae. pallidostriatus* and *Aedes* spp. 1 grouped into another clade with *Ae. ochraceus* and *Ae. vexans* sequences.

**Fig. 4:**
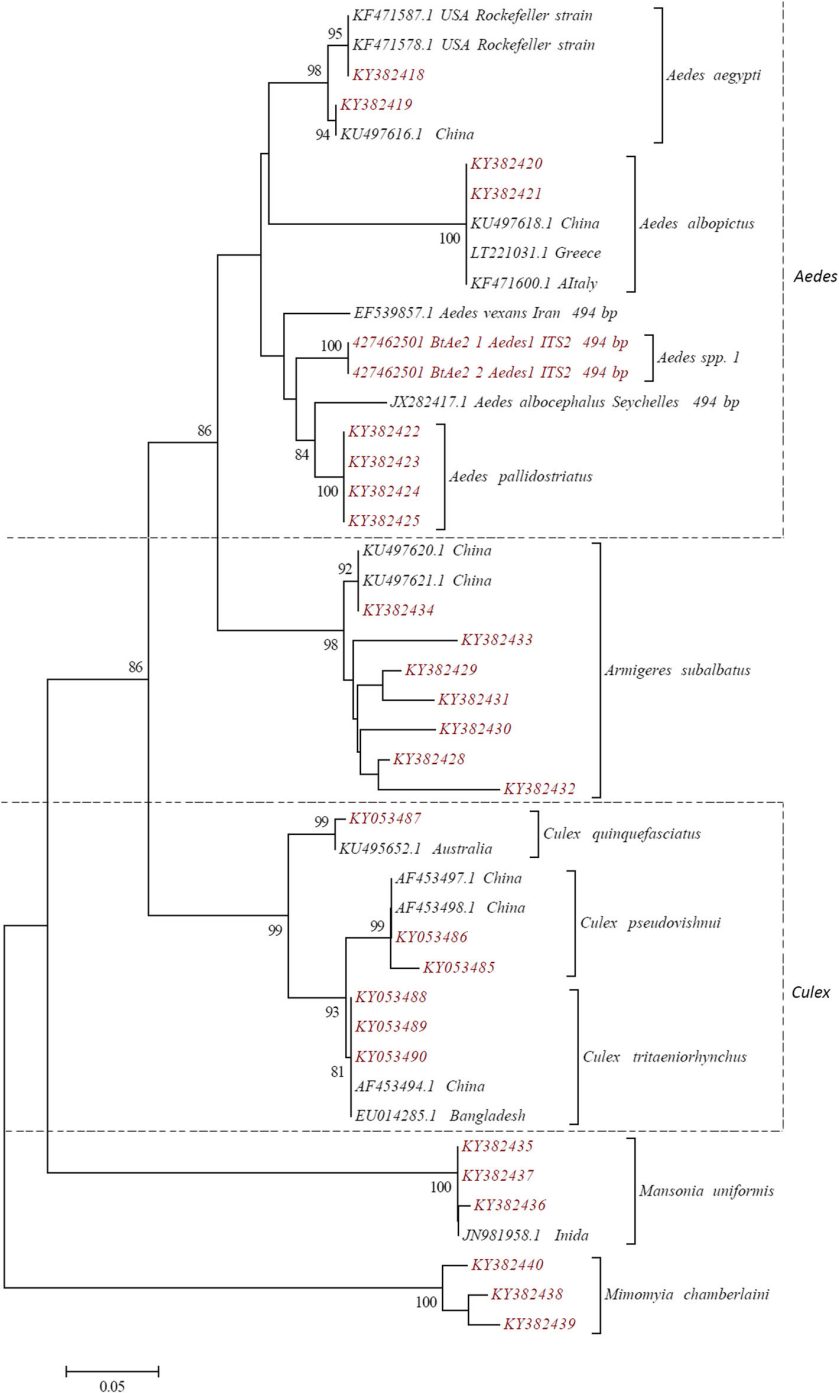
NJ phylogenetic tree based on Kimura 2-parameter genetic distance model for ITS2 sequences of all the 29 haplotypes of 10 species belonging to the subfamily Culicinae, collected from Sri Lanka during the study (red labels) and the sequences retrieved from the GenBank (back labels). Only nodal support > 70% is shown

Figure 4 shows the NJ tree constructed using the ITS2 sequences generated for the 10 mosquito species (except *Culex bitaeniorhynchus*, *Culex fuscocephala*, *Culex gelidus* and *Culex whitmorei*). All 10 species formed 10 strongly supported clades, each representing individual species. Further, 5 genera formed 5 major clades.

### DNA polymorphism analysis of *cox*1 sequences

*Cox*1 sequences were AT rich and the G+C content was 0.333. The number of variable bases was 145, accounting for 33.88% of the total number of sites. Among these variable sites, 142 were parsimony informative sites and only 3 represented singleton mutations. These nucleotide variations were heavily skewed to the third codon position which had 112 variable sites and 1 singleton mutation. First codon position had 28 variable sites and 2 singletons. The remaining 5 variable sites were at the second codon position without any singleton mutations.

*Aedes* sp. 1, *Cx. fuscocephala*, *Cx. pseudovishnui*, *Cx. quinquefasciatus* and *Cx. tritaeniorhynchus* had a haplotype diversity of 1.000, followed by *Ma. uniformis*. *Aedes albopictus* had the lowest haplotype diversity (0.600 ± 0.046) (Table 1).

As shown in Table 2, the mean intraspecific K2P distances for all the species were less than 2%. The maximum distance was seen among the haplotypes of *Cx. fuscocephala* which was 1.4% while *Ae. albopictus*, *Cx. quinquefasciatus* and *Mi. chamberlaini* reported the lowest mean intraspecific distance of 0.2%. The interspecific distances ranged from 6.8% between *Cx. whitmorei* and *Cx. tritaeniorhynchus* to 21.6% between *Ma. uniformis* and *Ae. albopictus* (Table 2).

**Table 2:** Interspecific (below the diagonal) and mean intraspecific distances (along the diagonal) for *cox*1 sequences. Distances were calculated using Kimura 2-parameter distance algorithm

	Species	1	2	3	4	5	6	7	8	9	10	11	12	13	14
1	*Ae. aegy*	0.010													
2	*Ae. albo*	0.147	0.002												
3	*Ae. pall*	0.151	0.144	0.009											
4	*Aedes* 1	0.140	0.143	0.095	0.003										
5	*Ar. suba*	0.145	0.150	0.138	0.133	0.004									
6	*Cx. bita*	0.156	0.148	0.133	0.146	0.153	0.000								
7	*Cx. fusc*	0.164	0.160	0.142	0.137	0.147	0.084	0.014							
8	*Cx. geli*	0.150	0.142	0.146	0.153	0.152	0.077	0.107	0.000						
9	*Cx. pseu*	0.184	0.163	0.132	0.142	0.144	0.114	0.101	0.102	0.009					
10	*Cx. quin*	0.129	0.138	0.116	0.138	0.144	0.086	0.086	0.087	0.109	0.002				
11	*Cx. trit*	0.140	0.145	0.117	0.146	0.135	0.090	0.101	0.086	0.086	0.077	0.007			
12	*Cx. whit*	0.153	0.134	0.129	0.128	0.144	0.079	0.092	0.071	0.086	0.069	0.068	0.000		
13	*Ma. unif*	0.183	0.216	0.164	0.162	0.197	0.198	0.205	0.181	0.196	0.181	0.175	0.185	0.008	–
13	*Mi. cham*	0.141	0.168	0.145	0.162	0.178	0.112	0.131	0.140	0.150	0.133	0.126	0.134	0.190	0.002

*Abbreviations*: *Ae*. *aegy* *Aedes aegypti*, *Ae. albo*, *Ae. albopictus*, *Ae. pall* *Ae. pallidostriatus*, *Aedes 1* *Aedes * sp. 1, *Ar. suba* *Armigeres subalbatus*, *Cx. bita* *Culex bitaeniorhynchus*, *Cx. fusc* *Cx. fuscocephala*, *Cx. geli* *Cx. gelidus*, *Cx. pseu* *Cx. pseudovishnui*, *Cx. quin* *Cx. quinquefasciatus*, *Cx. trit* *Cx. tritaeniorhynchus*, *Cx. whit* *Cx. whitmorei*, *Ma. unif* *Mansonia uniformis*, *Mi. cham* *Mimomyia chamberlaini *

## Discussion

The present study provides both morphological and molecular characterization of a collection of mosquitoes belonging to subfamily Culicinae in Sri Lanka for the first time. Using the traditional morphology-based taxonomy, 14 mosquito species belonging to the genera *Aedes*, *Armigeres*, *Culex*, *Mansonia* and *Mimomyia* were identified. Three *Aedes* species were identified to the species level: *Ae. aegypti*, *Ae. albopictus* and *Ae. pallidostriatus*. The species referred to as *Aedes* sp. 1 and *Armigeres* sp. 1 could not be identified into its species level due to physical damages.

Seven *Culex* species, i.e. *Cx. bitaeniorhynchus*, *Cx. fuscocephala*, *Cx. gelidus*, *Cx. pseudovishnui*, *Cx. quinquefasciatus*, *Cx. tritaeniorhynchus* and *Cx. whitmorei*, one *Mansonia* species, *Ma. uniformis*, and one *Mimomyia* species, *Mi. chamberlaini*, were recognized with the aid of taxonomic keys. DNA barcoding conducted with *cox*1 and ITS2 confirmed the identity of these species and the sequence similarity with the publicly available sequences in the GenBank and BOLD system was 99–100%, except for *Ae. pallidostriatus* and *Aedes* sp. 1. There were no available sequences for *Ae. pallidostriatus* and ours was the first GenBank record of this species. Although *Ae. vexans* showed the closest sequence similarity to *Aedes* sp. 1, its species identity could not be finalized using both molecular data and morphological identification. Although species status of *Armigeres* samples was not identified morphologically, sequence data clearly support its identification as *Ar*. *subalbatus*.

The NJ phylogenetic trees constructed using both *cox*1 and ITS2 sequences, and genetic distance analysis, further supported the species identity and displayed the phylogenetic relationships between the species. Each individual species was represented by well supported clades (> 98% bootstrap support) confirming the morphological identification of 14 species. Interspecific distance of more than 3% between the *cox*1 sequences is considered as the threshold in differentiating species [1, 34] and this has been applied in many mosquito phylogenetic studies [4–6, 13]. According to the genetic distance estimates of *cox*1 sequences, the intraspecific distances of all the species identified during the present study was less than 3% (ranged between 0.2–1.4 %) while the interspecific distances were above 3% (6.8–21.6%). Mosquito barcoding studies have previously reported divergence ranges similar to the present study [4–6, 13]. Ten of the mosquitoes with ITS2 sequences (except *Cx. bitaeniorhynchus*, *Cx. fuscocephala*, *Cx. gelidus* and *Cx. whitmorei*) were represented by more than one ITS2 haplotype. Previous studies, had also reported ITS2 haplotype variations within a single species [35–37]. However, a study on anopheline mosquitoes from Sri Lanka reported the presence of species-specific ITS2 haplotypes only [13].

*Aedes pallidostriatus* and *Aedes* sp. 1 showed highest sequence similarity to *Ae. ochraceus* and *Ae. vexans*, respectively. Based on the morphological features, *Ae. aegypti* and *Ae. albopictus* belong to subgenus *Stegomyia* while *Ae. pallidostriatus*, *Ae. ochraceus* and *Ae. vexans* belong to subgenus *Aedimorphus*. In the phylogenetic tree based on *cox*1 sequences, the two members of the subgenus *Stegomyia* formed a single clade while *Aedes* sp. 1 grouped with the members of the subgenus *Aedimorphus*. Therefore, the molecular data strongly suggest that *Aedes* sp. 1 is a member of *Aedimorphus*.

*Culex* species recognition is mainly based on morphology of adult females and fourth-instar larvae. However, absence and overlapping of morphological characters have often been identified as factors that lead to misidentification of *Culex* species [38]. Hence, molecular characterization is important in accurate and precise identification of them. The present study provides a basis for *Culex* species identification in Sri Lanka using molecular approach. The mean *cox*1 intraspecific distance ranged between 0.2–1.4% and the interspecific distance ranged between 7.0–11.2% for *Culex* species. Similar interspecific distances have been reported for *Culex* species from eastern Amazonian Ecuador [39] and India [8]. The intraspecific distances evaluated for 22 *Culex* species in Argentina and Brazil varied between 0.09–3.00% [38] and that for 13 *Culex* species in Turkey between 0–0.8% [40].

Accurate and precise identification of mosquito vectors and determination of their genetic diversity is important especially in determining the vectorial capacities and planning vector control strategies. Among the mosquitoes studied, *Ae. aegypti* and *Ae. albopictus* are the primary and secondary vectors, respectively, of dengue and chikungunya, *Cx. tritaeniorhynchus* is the major vector of Japanese encephalitis (JE) and *Cx. quinquefasciatus* major vector of filariasis in Sri Lanka. Also, JE virus has been isolated from wild-caught *Cx. pseudovishnui*, *Cx. gelidus*, *Cx. fuscocephala* and *Cx. whitmorei* from Sri Lanka [22], and these too were barcoded during the current study. According to our results, almost all the species tested showed high genetic diversity which, in turn, demands a greater attention since uniform control measures might not work in the same manner for all the populations of a single species. The present study highlights the importance of molecular characterization in species recognition of mosquitoes which can be successfully incorporated to future development and implementation of vector control strategies.

## Conclusions

The study showed that molecular characterization can be successfully employed for species identification of Culicinae mosquitoes. Results of DNA barcoding, using a combination of the genetic markers *cox*1 and ITS2, were comparable with morphological identifications and more importantly, DNA barcoding could accurately identify the species in the instances where the traditional morphological identification failed due to damaged specimens and indistinguishable characters. The *cox*1 and ITS2 sequences generated and submitted to the GenBank database could be used as reference sequences in future mosquito taxonomic studies. High genetic diversities observed in vectors of mosquito-borne diseases such as dengue, chickungunya and Japanese encephalitis should be taken into account in planning future vector control programmes in the country. Implementation of vector control programmes must be planned cautiously as uniform control measures may not be equally effective for genetically different populations.

## Additional file

Additional file 1: **Table S1.** Morphologically identified mosquito species, *cox*1 (fragment size of 428 bp) and ITS2 sequences (fragment sizes are separately listed for each species) generated from them, and the GenBank accession numbers for relevant submissions. Details of the closest publicly available sequences are also presented for comparison. (DOCX 19 kb)

## Abbreviations

BOLD: Barcode of Life Database
*cox*1: Cytochrome *c* oxidase subunit 1
ITS2: Internal transcribed spacer 2 region
K2P: Kumura-2 Parameter
NJ: Neighbor-Joining
