# Supplementary material for: DNA barcoding of morphologically characterized mosquitoes belonging to the subfamily Culicinae from Sri Lanka
Source: Parasit Vectors. 2018 Apr 25;11:266. doi: 10.1186/s13071-018-2810-z (PMC5918568; doi:10.1186/s13071-018-2810-z)
Supplement: Supplementary file 1 — Table S1. Morphologically identified mosquito species, cox1 (fragment size of 428 bp) and ITS2 sequences (fragment sizes are separately listed for each species) generated from them, and the GenBank accession numbers for relevant submissions. Details of the closest publicly available sequences are also presented for comparison. (DOCX 19 kb) [file 13071_2018_2810_MOESM1_ESM.docx]

**Additional file 1: Table S1.** Morphologically identified mosquito species, *cox*1 (fragment size = 428 bp) and ITS2 sequences (fragment sizes are separately listed for each species) generated from them, and the GenBank accession numbers for relevant submissions. Details of the closest publicly available sequences are also presented for comparison

| Species | *cox*1 | | | | ITS2 | | | | |
| --- | --- | --- | --- | --- | --- | --- | --- | --- | --- |
|  | ***n*** | **h** | **GenBank ID** | **Closest publicly available sequences** | ***n*** | **h** | **Fragment size (bp)** | **GenBank ID** | **Closest publicly available sequences** |
|  |  |  |  |  |  |  |  |  |  |
| *Aedes aegypti* | 4 | 3 | KY352243–KY352244, KY352256 | *Ae. aegypti*  (KX227741, KX227735) India | 3 | 2 | 335 | KY382418–KY382419 | *Ae. aegypti*  (KX227741, KX227735) India |
| *Ae. albopictus* | 6 | 3 | KY352245–KY352247 | *Ae. albopictus*  (KT260198, KT260200) India | 2 | 2 | 403 | KY382420–KY382421 | *Ae. albopictus*  (KU497618) China |
| *Ae. pallidostriatus* | 6 | 4 | KY352248–KY352251 | 92% *Ae. ochraceus*  (KJ940749, KJ940727) Kenya | 4 | 4 | 376 | KY382422–KY382425 | 90% *Ae. ochraceus*  (KJ940865–866) Kenya |
| *Aedes* sp. 1 | 4 | 4 | KY352252–KY352255 | 94% *Ae. vexans*  (KM452935) Hungary | 2 | 2 | 370 | KY382426–KY382427 | 82% *Ae. vexans*  (EF539857) Iran |
| *Armigeres* sp. 1 | 9 | 7 | KY352257–KY352263 | *Ar. subalbatus*  (KF564758, KF564760) Singapore | 8 | 7 | 392 | KY382428–KY382434 | *Ar. subalbatus*  (KU497620–621) China |
| *Culex bitaeniorhynchus* | 1 | 1 | KY040659 | *Cx. bitaeniorhynchus*  (KF564715) Singapore | – | – | – | – | – |
| *Cx. fuscocephala* | 2 | 2 | KY040660–KY040661 | *Cx. fuscocephala*  (DQ149236) India | – | – | – | – | – |
| *Cx. gelidus* | 2 | 1 | KY053491 | *Cx. gelidus*  (AY729965) India | – | – | – | – | – |
| *Cx. pseudovishnui* | 4 | 4 | KY040662–KY040665 | *Cx. pseudovishnui*  (KM350675) | 2 | 2 | 380 | KY053485–KY053486 | *Cx. pseudovishnui*  (AF453497–498) China |
| *Cx. quinquefasciatus* | 4 | 4 | KY040666–KY040669 | *Cx. quinquefasciatus*  (KM280579–80) Australia | 1 | 1 | 396 | KY053487 | *Cx. quinquefasciatus*  (KU495652) Australia |
| *Cx. tritaeniorhynchus* | 5 | 5 | KY040670–KY040674 | *Cx. tritaeniorhynchus*  (DQ424952) | 3 | 3 | 375 | KY053488–KY053490 | *Cx. tritaeniorhynchus*  (AF453494) China |
| *Cx. whitmorei* | 1 | 1 | KY040675 | *Cx. whitmorei*  (DQ154167) India | – | – | – | – | – |
| *Mansonia uniformis* | 11 | 9 | KY352264–KY352272 | *Ma. uniformis*  (KJ412468, AY729988) India | 8 | 3 | 380 | KY382435–KY382437 | *Ma. uniformis*  (JN981958) India |
| *Mimomyia chamberlaini* | 3 | 2 | KY352273–KY352274 | *Mi. chamberlaini*  (AY729979) India | 3 | 3 | 372 | KY382438–KY382440 | None |

*Abbreviations*: *n*, number of sequences; h, number of haplotypes
